# Antibiotic stewardship implementation at hospitals without on-site infectious disease specialists: A qualitative study

**DOI:** 10.1017/ice.2021.203

**Published:** 2022-05

**Authors:** Daniel J. Livorsi, Kenda R. Stewart Steffensmeier, Eli N. Perencevich, Matthew Bidwell Goetz, Heather Schacht Reisinger

**Affiliations:** 1 Center for Access & Delivery Research & Evaluation (CADRE), Iowa City Veterans’ Affairs (VA) Health Care System, Iowa City, Iowa; 2 Department of Internal Medicine, University of Iowa Carver College of Medicine, Iowa City, Iowa; 3 VA Greater Los Angeles Healthcare System, Los Angeles, California; 4 David Geffen School of Medicine at the University of California in Los Angeles, Los Angeles, California

## Abstract

**Background:**

Hospitals are required to have antibiotic stewardship programs (ASPs), but there are few models for implementing ASPs without the support of an infectious disease (ID) specialist, defined as an ID physician and/or ID pharmacist.

**Objective:**

In this study, we sought to understand ASP implementation at hospitals that lack on-site ID support within the Veterans’ Health Administration (VHA).

**Methods:**

Using a mandatory VHA survey, we identified acute-care hospitals that lacked an on-site ID specialist. We conducted semistructured interviews with personnel involved in ASP activities.

**Setting:**

The study was conducted across 7 VHA hospitals.

**Participants:**

In total, 42 hospital personnel were enrolled in the study.

**Results:**

The primary responsibility for ASPs fell on the pharmacist champions, who were typically assigned multiple other non-ASP responsibilities. The pharmacist champions were more successful at gaining buy-in when they had established rapport with clinicians, but at some sites, the use of contract physicians and frequent staff turnover were potential barriers. Some sites felt that having access to an off-site ID specialist was important for overcoming institutional barriers and improving the acceptance of their stewardship recommendations. In general, stewardship champions struggled to mobilize institutional resources, which made it difficult to advance their programmatic goals.

**Conclusion:**

In this study of 7 hospitals without on-site ID support, we found that ASPs are largely a pharmacy-driven process. Remote ID support, if available, was seen as helpful for implementing stewardship interventions. These findings may inform the future implementation of ASPs in settings lacking local ID expertise.

Antibiotic stewardship programs (ASPs) improve antibiotic prescribing and, in turn, reduce antibiotic resistance.^1^ Although a variety of resources are needed to implement hospital-based ASPs, the support of infectious disease (ID) pharmacists and physicians seems to be particularly important.^1^ The involvement of an on-site ID pharmacist and/or ID physician was a common element in several randomized-controlled trials that demonstrated the effectiveness of antibiotic stewardship.^2-6^ In addition, a cluster-randomized trial found that only community hospitals with the highest level of off-site ID support were able to achieve reductions in total antibiotic use.^7^ Finally, observational data from the Veterans’ Health Administration (VHA) demonstrated that patients at hospitals with on-site ID support received antibiotics in a way more consistent with stewardship principles than patients at hospitals without such support.^8^

Despite its benefits, many hospitals do not have access to ID expertise. Prior surveys have found that 41%–50% of US community hospitals lack an ID physician, whereas 93% lack an ID-trained pharmacist.^9,10^ In a 2016 survey of 244 US hospitals, the lack of ID or stewardship expertise was a common barrier to implementing prospective audit-and-feedback, which is one of the core stewardship strategies.^11^

It is unclear how hospitals without local ID support are implementing ASPs and whether these approaches are effective. In this VHA study, we sought to understand how antibiotic stewardship programs had been developed and maintained at VHA hospitals that lack on-site ID support.

## Methods

### Ethics

The Institutional Review Board (IRB) of the University of Iowa and Iowa City Veterans’ Health Care System approved this study. A waiver for written informed consent was granted.

### Site selection

This study was conducted within the national VHA system, which includes 130 acute-care hospitals. In 2014, the VHA mandated that all of its facilities develop and maintain an ASP.^12,13^ A key part of this directive was that each hospital was required to identify an antibiotic stewardship pharmacist and provider champion. Champions were expected to co-lead the ASP. In facilities without an ID physician, the provider champion could be a hospitalist, primary care provider, or advanced practice provider.

For this study, hospitals were eligible for inclusion if they had acute-care beds and lacked an on-site ID specialist. An ID specialist was defined as a pharmacist or physician who had completed a formal postgraduate residency or fellowship training program in ID. We identified eligible hospitals using data from a mandatory antibiotic stewardship survey, which had been administered by the VHA between December 30, 2015, and January 15, 2016, and was completed by a hospital staff member who was knowledgeable about local antibiotic stewardship activities.^14^ We have previously described the survey questions that informed whether we deemed a hospital to have an ID specialist.^8^

Once an eligible site was identified from the survey data, we used internal VHA resources to identify the designated antibiotic stewardship pharmacist and provider champion at each site. We contacted these champions by e-mail during March–April 2019 to confirm that the hospital still lacked an on-site ID specialist and to gauge their interest in participating in the study.

Of the 18 sites that met our inclusion criteria, 5 had hired an ID specialist since the survey was conducted and no longer qualified for the study. An additional 6 sites refused to participate and 7 sites were eligible and willing to participate.

### Site visits

In this study, 4 site visits were conducted in-person and, due to the COVID-19 pandemic, 2 visits were completed virtually. At the seventh site, only the ASP pharmacist champion agreed to be interviewed, so we chose to conduct a telephone interview rather than travel to the site. Data were collected between July 2019 and April 2020.

During the on-site and virtual visits, we attempted to interview all members of the ASP team, inpatient physicians who frequently interacted with the ASP team, and members of the hospital’s leadership. A medical anthropologist (K.S.S.) and an infectious disease specialist with qualitative training (D.J.L.) conducted all interviews.

Interviewers followed a semistructured interview guide (Appendices 1–4 online) with questions focused on the feasibility, appropriateness, and acceptability of implementing different antibiotic stewardship strategies.^15^ Interviews were recorded, transcribed, and reviewed for accuracy, except at the seventh site, where only notes were taken according to the lone participant’s request.

### Data analysis

We uploaded transcripts into MAXQDA, a management software program for qualitative data (VERBI Software, Berlin, Germany). We analyzed the data using thematic analysis; the code book was developed based on the interview guide (deductive) as well as interview responses and field notes (inductive).^16^ The analytic team (D.J.L. and K.S.S.) read 8 transcripts and shared their general impressions. Next, the analytic team independently coded transcripts, coding every third transcript in common. The team met weekly to review coded transcripts, discuss discrepancies, and reach consensus. After coding was complete, the team reviewed how each code intersected with the code for barriers or the code for facilitators. After reviewing the groups of coded segments separately, the 2 analysts shared their findings and identified common themes.

## Results

Table 1 shows characteristics of the 13 hospitals that lacked an on-site ID specialist, including the 7 hospitals we visited and the 6 that refused to participate. Across all 13 sites, 7 (54%) were rural. The professional roles of all 42 interviewees are shown in Table 2.

**Table 1.** Characteristics of 13 VHA Hospitals That Lacked an On-Site Infectious Diseases Specialist in 2019, Stratified by Their Willingness to Participate in this Study

Characteristic	Agreed to Participate (n=7), No. (%)	Refused to Participate (n=6), No. (%)
**US census region**		
Northeast	2 (29)	1 (17)
Midwest	1 (14)	3 (50)
South	3 (29)	2 (33)
West	1 (14)	0
Rural location	3 (43)	4 (67)
**Size, median (IQR)**		
Acute-care beds	18 (14–30)	27 (16–29)
Community living center^a^	55 (40–85)	99 (46–158)
Intensive care unit	3 (43)	3 (50)
**VHA complexity level^b^**		
1c	2 (29)	1 (17)
2	2 (29)	3 (50)
3	3 (43)	2 (33)
**FTEEs for antibiotic stewardship**		
0	5 (71)	
0.25	1 (14)	…^c^
1	1 (14)	

Note. FTEE, full-time employment equivalent; IQR, interquartile range; VHA, Veterans’ Health Administration; CLC, community living center.

a CLCs are like skilled nursing facilities. These were present at 6 of the 7 sites, and the median only reflects the sites that had a CLC.

b The VHA categorizes its medical centers by complexity: 1a, 1b, 1c, 2, or 3. The complexity level reflects a medical center’s patient population, services provided, and resources for education and research. The least complex centers are categorized as level 3.

c Because these sites were not visited, we were unable to collect data on FTEEs.

**Table 2.** Characteristics of 42 Participants in Semistructured Interviews at 7 VHA Hospitals

Role	No. of Participants
Clinical pharmacy specialists	10
Hospitalists	9
**Administrators**	
Pharmacy	6
Chief of medicine	2
Associate chief of staff	1
Microbiologist	5
Off-site ID physician	2
Emergency department/Urgent care physician	2
Nurse practitioners^a^	2
Other^b^	2
CLC physician	1

Note. VHA, Veterans’ Health Administration; ID, infectious disease; CLC, community living center.

a One nurse practitioner worked in the community living center and the other worked in acute care.

b Other includes 1 podiatrist and 1 infection preventionist.

All 7 sites had a designated pharmacist champion, and 6 (86%) sites had named an ASP provider champion. At least 1 ASP pharmacist champion per site had completed an antibiotic stewardship certification course (ie, a course put on by either the Society of ID Pharmacists or Making a Difference in ID). At 6 sites (86%), the primary responsibility of the ASP pharmacist champions was to provide clinical pharmacy services to a specific area of the hospital, such as acute care (3 sites), the community living center (CLC; 2 sites) or the emergency department (ED; 1 site). The primary role of the provider champions was as follows: hospitalist physician (2 sites), hospital administrator (2 sites), hospitalist advanced practice provider (1 site), and geriatrician (1 site).

All sites prepared annual antibiograms and used education and some form of prospective audit-and-feedback to improve inpatient antibiotic-prescribing. Five sites (71%) reported having inpatient antibiotic-prescribing orders sets. Several sites were also working to improve outpatient antibiotic use, largely through education and retrospective audit and review.

Although sites reported using similar interventions to promote antibiotic stewardship, the shape of those interventions varied. The following 4 themes illustrate the ways in which hospitals without on-site ID support had implemented ASPs and the barriers they encountered (Table 3).

**Table 3.** Sample Quotations from Semistructured Interviews With 42 Hospital Personnel Involved in Antibiotic Stewardship Activities Across 7 VHA Hospitals That Lacked an On-Site Infectious Disease Specialist

Hospital Personnel	Interview Quote
**Theme 1. ASP pharmacist champions wear many hats and fill many roles**	
ASP pharmacist champion, site 1	“I was starting to get overwhelmed with all the requirements because I also do, you know, the IV program and a lot of training and inpatient and stewardship and it just, it’s too much for one person, essentially.”
ASP pharmacist champion, site 2	“There’s a lot of things that come down from national, and there’s only so many people to do them. I had a very good relationship with our pharmacy manager at the time. I can remember the day when we were standing at the back of the pharmacy…. My boss just was talking about, ‘Well, we have to cover this now,’ and I think maybe even she said, ‘Well, you guys use a ton of antibiotics [in the CLC] or whatever and… so, I guess it’s going to be me then.’”
ASP pharmacist champion, site 4	“In this kind of smaller facility, you don’t have a lot of the different specialties or things you would normally have in some of the bigger facilities, so you end up kind of being a kind of jack of all trades.”
**Theme 2. Achieving stewardship buy-in takes time and is influenced by patterns of clinician staffing**	
ASP pharmacist champion #1, site 1	“I guess I feel when you have a face-to-face, you get to know somebody. You get to have trust in them and, I mean, one of our providers, it took him probably a year to ever come to me and ask a question because I had to prove that I was worthy of his time…. Now he’ll come to me with things.”
Inpatient clinical pharmacy specialist, site 3	“We used to have a lot of turnover [with the hospitalists], so you didn’t get to know them as well and develop a relationship with them. But this group has been here for a while now. So I think they know it’s coming…. If it’s something they don’t want to do or they don’t agree with, they’re pretty good about talking to me about it and saying, ‘Look. This is why I don’t want to do that.’ And, you know, we have good conversations about it.”
ASP physician champion, site 2	“But then there’s this whole host of contracted group [physicians] that comes in and there’s a lot of different people that potentially could be coming in and out. And that’s a real struggle with our efforts as you can’t get these guys all on one place at one time to have a conversation. You know, they come, and they go and so the people you talk to in this month, may be different than the people that are here working 2 months from now. So you kind of reinvent the wheel all the time. Their level of engagement and interest is…not the best. They’re here just working on an hourly basis. Like a locums doctor and filling in and so they’re not really real committed to the bigger picture items.”
**Theme 3. Having access to an off-site ID specialist improved the local acceptance of stewardship efforts**	
ASP pharmacist, champion #2, site 1	“Having an ID physician on-site would allow us to probably make more protocols that would get accepted and make more treatment guidelines for physicians to use as tools, especially in our outpatient setting where we’re staffed with a lot of mid-levels. We don’t have a lot of actual MDs that are working, and so, those tools are very useful for them.”
ASP pharmacist champion, site 3	*[What if the remote ID physician was not involved?]* “It’d probably be a little harder to get some things passed. I guess I’d probably have to just make a fight and justification going up the committees and all of that. That would be the biggest thing.”
Pharmacy administrator, site 7	“Bless, bless those folks [at the other VA hospital within our VISN]…. They actually have an ID clinical pharmacy specialist, and she is very helpful [to us].”
ASP pharmacist champions, site 1	“I think he [the non-ID ASP physician champion] is more than willing to help us out in that respect [when we are encountering resistance from physicians]. I feel bad asking him to do that sometimes because … he’s not ID. He’s just our physician champion, which is great. We appreciate it, but I feel like sometimes we don’t trump it because we can’t say, ‘Our ID physician recommends it.’”
Hospitalist, site 1	“I find that the person [remote ID physician] that we’ve partnered with is probably the most talented clinical teacher over the phone.… He’s a super good resource. I think part of that is the durability of those relationships over years and years and years and years. It’s a lot different than calling somebody that you don’t know, that’s carrying a pager and giving advice.”
ASP pharmacist champion, site 2	“I think from the perspective of being the stewardship pharmacist, but not someone who went through Infectious Disease training, having those more in depth discussions with the infectious disease physician has always been pretty good learning experience for me.”
**Theme 4. Access to institutional resources is an important driver of ASP success**	
ASP pharmacist champion, site 4	“They [the hospital leadership] gave me more time [ie, FTEs] when we were due for a Joint Commission survey to try and at least kind of get some boxes checked, so to at least minimally satisfy the surveyors.”
Inpatient clinical pharmacist, site 3	“If you want to build an order set, it has to go through 20 people above you and then this CAC [clinical applications coordinator] left that was very helpful to us…. That probably eats up a ton of his [the ASP pharmacist champion’s] time. Whereas [at a hospital with information technology support] he could just say, ‘Here. This is the way I want it to be. Put it in place.’”
ASP pharmacist champion, site 1	“Two and half, three years ago we tried for a dedicated FTE…. We wrote up a business proposal and presented it to physicians and chief of staff and all that and talked to the clients, and … it’s like it evaporated in thin air…. It was green lit then and then afterward, like something happened and they said, ‘No.’”
Laboratory Director, site 1	“They haven’t funded stewardship in pharmacy…. We’re like barely meeting the minimum, and I told [the ASP pharmacy champion] I would really love for us to fail on Joint Commission or something like that. And then that would make them have to do something.”

Note. ID, infectious disease; CLC, community living center; VHA, Veterans’ Health Administration.

### Theme 1. ASP pharmacist champions wear many hats and fill many roles

At 6 sites, being the ASP pharmacist champion was an added responsibility to their primary pharmacist role. Only 1 site had an ASP pharmacist champion whose only responsibility was antibiotic stewardship.

Most of the ASP pharmacist champions spoke positively about filling their many roles. One ASP pharmacist champion explained: “One of the benefits of working at a small site is you wear lots of hats… It’s a good professional challenge for me” (site 7). Several others expressed having a passion for stewardship, sometimes even remembering the exact moment they agreed to take on the job. According to one champion, “Not many pharmacists like bugs and drugs, [but] for some reason that’s always been one of my passions” (site 3).

Despite having several other responsibilities, the ASP pharmacist champion was consistently recognized as the daily leader of the program. Only a few sites had an engaged on-site ASP provider champion. At one site, the ASP pharmacist champion had been in place since the program’s inception but there had been 3 different provider champions during that same time. She complained, “Stewardship is seen as a pharmacy thing and not an everybody thing” (site 5).

### Theme 2. Achieving stewardship buy-in takes time and is influenced by patterns of clinician staffing

ASP pharmacist champions faced several challenges when trying to provide antibiotic recommendations to clinicians. Pharmacists recognized the importance of bedside patient care to informing antibiotic decisions, and they acknowledged the legal responsibility of the clinicians to manage patient care. Additionally, one ASP pharmacist champion said, “I have to build rapport with them to get them to listen to me but I have no recourse if they don’t…. I make a lot of suggestions and hope a couple of them stick” (site 4).

To help develop rapport with clinicians, pharmacists spoke of the importance of having face-to-face conversations, providing evidence to support stewardship recommendations, and being persistent. The importance of taking a collaborative approach to stewardship was also well recognized. As one ASP pharmacist champion said, “I want it to be a positive approach, and I don’t want it to be condescending in any way to anybody that I talk to about it [antibiotic stewardship]” (site 6).

Pharmacists who worked with clinicians on a range of clinical issues felt like these working relationships helped to build trust and specifically improved the physicians’ acceptance of their antibiotic recommendations. One ASP pharmacist champion whose primary role was in the ED explained, “ED physicians are very open to help especially when it comes to medications because they have so much going on at the same time. If I can say, ‘Hey, yeah, I can put in this medication,’ or I just tell them what to use, what dose, they really just love that support. So I think just kind of being down there [helps]” (site 3).

In some situations, working at a smaller hospital facilitated good rapport, especially when a consistent group of physicians was staffing the facility. According to one ASP pharmacist champion, “That’s the nice thing with it being a smaller facility. I’m not talking to 20 physicians … I’m talking really to 5 … as far as the hospitalists and CLC go, I’m talking to 5 providers for the most part” (site 4).

ASP pharmacist champions struggled to establish rapport with clinicians when a hospital relied heavily on contract physicians. One ASP pharmacist champion explained, “In the last year, we’ve had so many locums that it’s hard to track anything they do. There’s so many, a different person every week. We just got back in the last month to our standard [hospitalists], who have both been the hospitalists here for quite some time … and so it’s been nice to have a little bit of normalcy again” (site 6). Other sites struggled with the frequent turnover of outpatient providers and hospital leadership.

### Theme 3. Having access to an off-site ID specialist improved the local acceptance of stewardship efforts

At the sites we visited, a common sentiment was that the hospital was too small to justify an on-site ID specialist. All sites had access to ID physician electronic consultation for challenging cases, and 4 sites had access to an off-site ID specialist who assisted with at least some local ASP activities (sites 1, 2, 3, and 7). These remote ID specialists helped give credibility to both the ASP pharmacists’ recommendations and to any stewardship initiatives the ASP team was trying to move forward. According to the ASP pharmacist champion at site 2, who was discussing prospective audit and feedback, “I’ve found that having the support of an infectious diseases physician really makes a huge difference in terms of acceptance of the recommendations, particularly if it’s to stop antibiotic therapy. So maximizing access to that, I think, is something that we need.” An off-site ID specialist could also help overcome institutional barriers to putting new protocols or order sets into place. At site 5, the ASP pharmacist champion would “love to have ID physician support” for stewardship but acknowledged that, given the size of the hospital, it would be “hard to justify.”

The stewardship champions at 2 other sites (sites 4 and 6) did not perceive lack of ID support as a barrier to stewardship efforts. At site 4, the ASP pharmacist champion found it less helpful to consult with ID pharmacists at other sites as his facility was “very different” with a “completely different patient demographic.” However, a hospitalist at site 4 felt that ID consultation would have facilitated the application of stewardship principles: “At least from my standpoint, I can say ‘I consulted with infectious disease’ and then pass the buck there, but I can’t say, ‘Oh I consulted with pharmacy’ and then be totally left off the hook.” At site 6, the ASP provider and pharmacist champions had been working together for years. As the lead hospitalist, the provider champion was able to garner institutional buy-in for the ASP by demonstrating the overlap between excessive use of parenteral antibiotics and the metrics the hospital leadership cared about, such as length of stay and *Clostridioides difficile* infection.

For ASPs that were using remote ID specialists, the manner in which this expertise was accessed varied. One hospital had an informal arrangement with an off-site ID VHA physician to conduct prospective audit and feedback with the on-site ASP pharmacist champion twice per week (site 2). At the time of our visit, this arrangement was becoming more formalized due to the ID physician’s need to capture her workload credit. Two other hospitals sought support as needed from either an ID-trained physician or ID pharmacist at another VHA site within their VISN (Veterans’ Integrated Service Network). At these 2 sites, ID support was sought either to provide feedback on current antimicrobial therapy in specific patients or to provide input on new stewardship protocols that were under development. Finally, at one hospital (site 1) an off-site ID physician received 0.2 full-time employment equivalent (FTEE) to provide remote ID support to the hospital’s physicians, infection control department, and ASP. This off-site ID physician clearly added value, as one hospitalist explained: “I think that the [remote ID physician’s] work with the lab has really improved the confidence in the information that’s received.” At the time of our visit, the ID physician had recently retired, and there was nobody to fill this void. The newly appointed ASP provider champion, who was a hospitalist, acknowledged that his lack of ID training made it difficult for him to audit and provide feedback on the antibiotic prescribing of his colleagues: “Since I don’t have any really additional training compared to my hospitalist colleagues, I try to stay out of their cases and so it’s not really appropriate for me to be trying to intervene on their management.”

### Theme 4. Access to institutional resources is an important driver of ASP success

Most champions spoke of the importance and the difficulty of gaining institutional buy-in for their ASP. Hospital personnel were not always receptive to stewardship initiatives, and a common sentiment was that the ASP existed only to meet regulatory requirements (eg, the VHA directive and the Joint Commission mandate). It was challenging for the ASP champions to get further guidance on how their efforts could assist the facility. In the words of one ASP pharmacist champion, “I would say our facility expects us to pass our new requirements. To have Joint Commission walk in and give us a thumbs up and walk out. That’s what our leadership wants” (site 7). An ASP provider champion at another site echoed this frustration, “I’ve stood in front of the medical staff multiple times and asked for their input on what they’d like to see from the [stewardship] program and what do they think about this and that, and it’s like crickets. Nobody says a word” (site 2).

Lack of funding and lack of information technology support further hampered ASP efforts. However, funding a stewardship position was not a silver bullet, as shown by the one hospital that had assigned a full FTE to the ASP pharmacy champion: “I’ve got my FTE so I feel like a lot of the facility just thinks I’m just going to do it, but you know it’s hard for me to actually enact things…especially when we’ve had some turnover… I’ve had good advocates and then they leave” (site 4).

Some ASPs reported success engaging leadership to overcome specific barriers. For example, at one hospital, there was a contract physician who wanted to frequently prescribe ceftaroline. When the ASP pharmacist champion went to the chief of medicine, she was able to gain support for changing this specific physician’s practice (site 5). At another hospital, the chief of staff authorized a peer review to be performed on a specific hospitalist with consistent guideline-discordant antibiotic prescribing; once this was done, the hospitalist agreed to change his practice (site 3).

## Discussion

In this qualitative study of 7 hospitals without local ID support, we found that ASPs are largely an underfunded pharmacy-driven process. For some programs, having access to an off-site ID specialist was perceived to improve implementation of stewardship interventions, but other ASPs did not feel that the lack of this expertise was a barrier to their efforts.

In many ways, our findings at hospitals without on-site ID support are in line with prior qualitative work on ASPs at hospitals with ID support. At least 1 other US study of 46 hospitals reported that pharmacists were generally the leaders of day-to-day stewardship activities.^17^ Furthermore, like other studies, we found that ASP teams found that collaborative, nonjudgmental approaches to stewardship were more effective and better received by clinicians.^17-19^ An organizational culture in which the full clinical team collaborates and is supportive of stewardship is also essential.^20^

Our qualitative study is exceptional in that it only included hospitals without local ID support. As described elsewhere, we found that personnel at these small, often rural, hospitals typically fulfill multiple different roles and struggle with the high turnover of clinical staff.^21,22^ Also, some hospitals valued regular access to outside ID expertise for ASP support. As shown in our study, hospitals can access remote ID expertise through a variety of means, including part-time contracts, their health system network, or telehealth.^23^ Although the Centers for Disease Control and Prevention acknowledges a benefit to having ID specialists involved in ASPs, they also recognize that non-ID personnel can be effective leaders of stewardship efforts at smaller hospitals.^24^ A few reports support this recommendation.^25-27^

The findings from our work have broader implications, as a large proportion of US hospitals lack an on-site ID specialist but, based on regulatory requirements, are still expected to have an ASP. These hospitals, which tend to be small in size and rural in location, have been shown to have antibiotic use that is similar, if not higher, than hospitals with ID support.^8,28^ Ultimately, these hospitals will need to tailor their antibiotic stewardship processes according to their local needs, resources and personnel.^29^

Our study has a several limitations. First, we only visited sites that were willing to participate in our study, and our findings may therefore have been biased toward hospitals that had active ASPs willing to share their success or those who were especially struggling and viewed participation as a possible avenue for help. Second, it is unknown whether the experience of these VHA hospitals is generalizable to nonintegrated healthcare systems.

In conclusion, our study has provided a unique perspective on ASP implementation at 7 hospitals that lacked local ID support. Although many sites valued remote ID support for implementing stewardship processes, other sites did not feel that this expertise was necessary. Given the ongoing need to improve antibiotic use and expand the implementation of ASPs across the spectrum of healthcare, our findings could inform future work on ASP implementation in these settings.
